# Reappraising the concept of massive transfusion in trauma

**DOI:** 10.1186/cc9394

**Published:** 2010-12-30

**Authors:** Simon J Stanworth, Timothy P Morris, Christine Gaarder, J Carel Goslings, Marc Maegele, Mitchell J Cohen, Thomas C König, Ross A Davenport, Jean-Francois Pittet, Pär I Johansson, Shubha Allard, Tony Johnson, Karim Brohi

**Affiliations:** 1 NHS Blood & Transplant, Oxford Radcliffe Hospitals Trust, John Radcliffe Hospital, Headley Way, Headington, Oxford, OX3 9BQ, UK; 2 Medical Research Council (MRC), Clinical Trials Unit, 222 Euston Road, London, NW1 2DA, UK; 3 Department of Traumatology, Division of Critical Care, Oslo University Hospital Ulleval, Kirkeveien 166, 0407 Oslo, Norway; 4 Trauma Unit, Department of Surgery, Academic Medical Center, University of Amsterdam, Meibergdreef 9, 1105 AZ Amsterdam, The Netherlands; 5 Department of Traumatology and Orthopedic Surgery, Institute for Research in Operative Medicine (IFOM), Cologne-Merheim Medical Center (CMMC), University of Witten/Herdecke, Campus Cologne-Merheim, Ostmerheimerstr. 200, 51109 Cologne, Germany; 6 Department of Surgery, San Francisco General Hospital, University of California San Francisco (CA), Campus Box 0807, San Francisco, CA 94143-0807, USA; 7 Trauma Clinical Academic Unit, Barts and the London School of Medicine & Dentistry, Queen Mary, University of London, Mile End Road, London, E1 4NS, UK; 8 Departments of Anesthesiology and Surgery, University of Alabama at Birmingham, 804 Jefferson Tower, 619 South 19th Street, Birmingham, AL 35249-6810, USA; 9 Capital Region Blood Bank, Rigshospitalet, University of Copenhagen, Blegdamsvej 9, 2100 Copenhagen, Denmark; 10 NHS Blood & Transplant/Barts & London Trust, The Royal London Hospital, Whitechapel Road, Whitechapel, London, E1 1BB, UK; 11 MRC Biostatistics Unit, Institute of Public Health, University Forvie Site, Robinson Way, Cambridge, CB2 0SR, UK

## Abstract

**Introduction:**

The massive-transfusion concept was introduced to recognize the dilutional complications resulting from large volumes of packed red blood cells (PRBCs). Definitions of massive transfusion vary and lack supporting clinical evidence. Damage-control resuscitation regimens of modern trauma care are targeted to the early correction of acute traumatic coagulopathy. The aim of this study was to identify a clinically relevant definition of trauma massive transfusion based on clinical outcomes. We also examined whether the concept was useful in that early prediction of massive transfusion requirements could allow early activation of blood bank protocols.

**Methods:**

Datasets on trauma admissions over a 1 or 2-year period were obtained from the trauma registries of five large trauma research networks. A fractional polynomial was used to model the transfusion-associated probability of death. A logistic regression model for the prediction of massive transfusion, defined as 10 or more units of red cell transfusions, was developed.

**Results:**

In total, 5,693 patient records were available for analysis. Mortality increased as transfusion requirements increased, but the model indicated no threshold effect. Mortality was 9% in patients who received none to five PRBC units, 22% in patients receiving six to nine PRBC units, and 42% in patients receiving 10 or more units. A logistic model for prediction of massive transfusion was developed and validated at multiple sites but achieved only moderate performance. The area under the receiver operating characteristic curve was 0.81, with specificity of only 50% at a sensitivity of 90% for the prediction of 10 or more PRBC units. Performance varied widely at different trauma centers, with specificity varying from 48% to 91%.

**Conclusions:**

No threshold for definition exists at which a massive transfusion specifically results in worse outcomes. Even with a large sample size across multiple trauma datasets, it was not possible to develop a transportable and clinically useful prediction model based on available admission parameters. Massive transfusion as a concept in trauma has limited utility, and emphasis should be placed on identifying patients with massive hemorrhage and acute traumatic coagulopathy.

## Introduction

Hemorrhage is responsible for more than 40% of all trauma deaths and therefore represents an important target for improving outcomes after severe injury. The concept of massive transfusion has existed for more than half a century and was developed to highlight the dilutional complications occurring when administering large volumes of packed red blood cells (PRBCs) or other fluids, which could be addressed by the use of massive-transfusion protocols. Such protocols are not immediately activated but typically require either the presence of abnormal laboratory tests of coagulation [1,2] or the prior administration of a certain number of units of PRBCs [3].

It is now clear that standard massive-transfusion algorithms are less effective in trauma hemorrhage [4,5]. Primarily, this is due to the presence of an endogenous coagulopathy very early in the clinical course of trauma patients, due to the presence of shock and tissue hypoperfusion [6]. This acute traumatic coagulopathy (ATC) may be established by the time the patient arrives in the emergency department [7-10] and is strongly associated with the need for large volumes of blood transfusion [10]. New damage-control resuscitation protocols targeted at ATC call for earlier plasma and blood-component regimens [11], and significant improvements in outcome may be achievable with such strategies [12-14].

In the absence of validated near-patient diagnostic tools for ATC, some centers are moving to empiric transfusion protocols activated early on the basis of clinical judgment [3]. Prediction models for massive transfusion have been developed in both civilian [15-17] and military [18-20] settings, although in general, these published tools have only moderate performance. In clinical use, where sensitivity rates of more than 90% would be important, these tools have very low specificities of around 50%. These models were developed in specific populations and remain largely unvalidated outside of their original datasets.

We designed this international multicenter study to reappraise the utility of massive transfusion as a clinical concept in modern trauma care. The first aim of the study was to assess whether a clinically relevant definition of massive transfusion existed in terms of a clinical outcome. The second aim was to assess by predictive modeling whether transfusion therapy can be rapidly and appropriately instituted by using parameters potentially available on trauma center admission.

## Materials and methods

Datasets on trauma admissions were obtained from the trauma registries of a research network of major trauma centers. Participating trauma centers were the Royal London Hospital, London, UK; Oslo University Hospital Ulleval, Norway; Academic Medical Centre, Amsterdam, the Netherlands; and San Francisco General Hospital, San Francisco, California, USA. Data from The Trauma Registry of the Deutsche Gesellschaft für Unfallchirurgie (TR-DGU) [21,22] from Germany, which covers more than 100 hospitals, were also included. The datasets included information over a 1-year period (2007) except the Oslo dataset covering 2 years (from June 2005). The data included patient age, sex, penetrating injury (yes/no), time from injury to emergency department arrival, admission systolic blood pressure, base deficit, prothrombin time (PT) and Injury Severity Score (ISS) [23], number of packed red blood cells (PRBCs) transfused in the first 24 hours, and in-hospital or 30 day (Oslo) mortality. The authors confirm that each trauma registry of the network is approved by a local review board and is in compliance with the institutional and/or national legal frameworks and data-protection requirements. Informed consent was not required, according to institutional, local and national guidelines. All data collection and analysis was performed anonymously.

A fractional polynomial was used to relate the odds of death to PRBCs received by logistic regression; these polynomials allow great flexibility by combining combinations of integer powers (such as squares and cubes) and noninteger powers such as one-half (square root), one third (cubic root), and others.

We then developed a logistic regression model for the prediction of massive transfusion, defined as 10 or more units of PRBCs. Missing data were a problem and were dealt with by using multiple imputation by chained equations [24,25] under the assumption of missing at random [26]. Fifty imputed datasets were created (since time to emergency department was unobserved in 42% of patients) by using predictive mean matching, retaining imputed values obtained after 100 cycles. The imputation model was specified to be at least as complex as the prognostic model [27], including all candidate predictors. Normalizing transformations of the observed continuous variables were taken so that the distributions of imputed and observed values were similar. All candidate predictors potentially available on admission and thought to be associated with transfusion were considered. Center-specific effects were excluded to allow generalizability of results. Model parameters were estimated by combining across imputed datasets [28]. Backward elimination was used to select variables, with *P *> 0.1 as the elimination criterion. A shrinkage factor was applied to log odds ratios after model fitting before validation [29]. The same model was also fitted by using complete data without any imputation, to assess for any effects of imputation. The results were consistent with the multiple-imputation analysis, although the parameters were estimated with greater precision with imputation (data not shown). The Amsterdam data were not included in this complete analysis without imputation, because time to emergency department was not recorded at this center.

Two training-validation dataset scenarios were used. First, TR-DGU data from Germany were used for external validation [30], with all other data used for training. The German TR-DGU registry data contributed 1,705 patients, 30% of the total dataset, and was considered to be of a suitable size for validation. Further, no data were missing. As a second (internal) validation, data were split randomly with 60% of patients from each center in the training dataset and 40% in the validation dataset. Calibration [31] and receiver operating characteristic (ROC) plots were examined, along with sensitivity and likelihood ratio, at 90% specificity. The calibration plot was formed by predicting the likelihood of massive transfusion for each patient in the validation dataset [32]. Individuals were then grouped by predicted probability, and these groups were compared with the observed transfusions received. After validation, the model was evaluated with the full dataset. We examined between-center variation in the performance of the model to investigate the effect of center-specific transfusion practices. For these purposes, the model including variables chosen from the previous two analyses was fitted, and the predictive value was tested in each center separately to see how variable this was. All statistical analyses and graphics were produced in Stata version 10.1 (StataCorp, 4905 Lakeway Drive, College Station, TX, USA).

## Results

**Figure 1:**
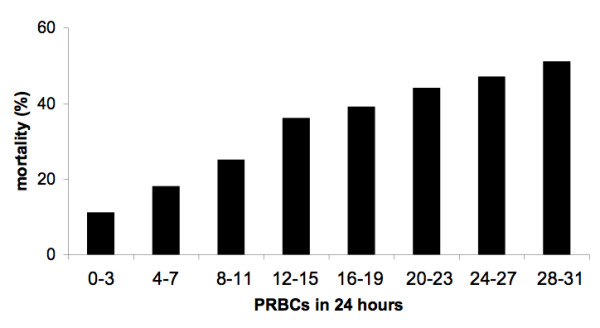
**Transfusion-related mortality**. Mortality by packed red blood cells (PRBCs) administered during the first 24 hours of admission.

**Figure 2:**
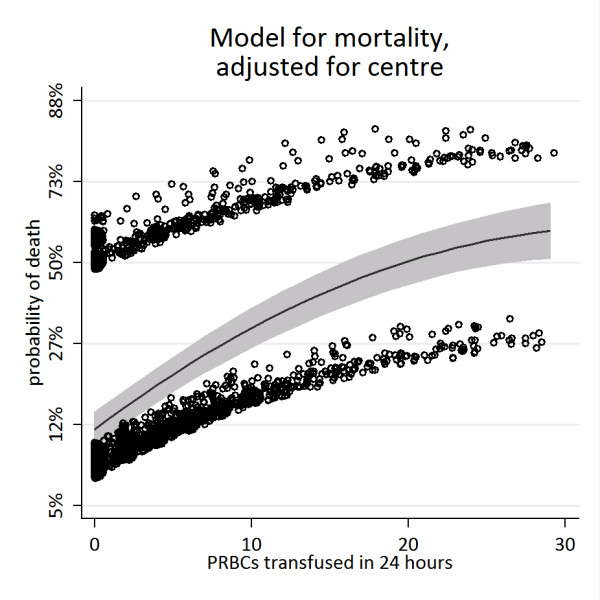
**Estimated probability of death per unit of packed red blood cells (PRBCs) administered (95% confidence interval in grey)**. Dots are deviance residuals. The band of dots above the line represents patients who died; the band below is those who survived.

In total, 5,693 patient records were available for analysis. Patient demographics, injury characteristics, admission physiology, base deficit, and prothrombin times are shown in Table 1. Records of 2,497 (44%) patients had a complete set of observed covariates, whereas one covariate was missing in 1,788 (31%) and two (14%) in 850. Mortality increased as transfusion requirements increased (Figure 1). No threshold effect was seen at 10 units or any other value of PRBC transfusions. Mortality was 426 (9%) of 4,808 in patients who received none to five PRBC units, 82 (22%) of 367 in patients receiving six to nine PRBC units, and 217 (42%) of 518 in patients receiving 10 or more PRBC units. The fractional polynomial model for transfusion-associated probability of death, adjusting for any institution effect, is shown in Figure 2. The open dots above and below the fitted line (deviance residuals) represent patients who died (above) and survived (below). These serve to illustrate that transfusion for patients who died and survived extends over the range of PRBC transfusions up to 30. The model did not demonstrate any steps or plateaus: each additional unit of blood transfused was associated with an increased risk of death.

**Table 1:** Demographics

	Number missing	All patients (*n *= 5,693)	London (*n *= 788)	Oslo (*n *= 2167)	San Francisco (*n *= 384)	Amsterdam (*n *= 649)	TR-DGU (*n *= 1705)
Massive transfusion cases (%)	0	518 (9%)	69 (9%)	68 (3%)	47 (12%)	12 (2%)	322 (19%)
Age in years (range)	24 (0.4%)	36 (24 to 53)	33 (24 to 46)	34 (21 to 51)	40 (26 to 56)	33 (20 to 49)	41 (27 to 58)
Male (%)	0	4,161 (73%)	636 (81%)	1,539 (71%)	294 (77%)	451 (69%)	1,241 (73%)
Penetrating injury (%)	23 (0.4%)	580 (10%)	150 (26%)	177 (8%)	125 (22%)	29 (5%)	99 (17%)
Injury Severity Score (range)	86 (2%)	17 (9 to 29)	16 (6 to 26)	12 (5 to 22)	18 (10 to 29)	5 (1 to 15)	27 (18 to 38)
Systolic blood pressure, mean (SD) (mm Hg)	425 (7%)	126 (29)	127 (30)	130 (32)	130 (32)	138 (26)	116 (29)
Base deficit, mean (m*M*, range)	865 (15%)	2.3 (0.2 to 5.3)	2.6 (0.4 to 6.2)	1.2 (-0.6 to 3.4)	5.5 (3.0 to 9.3)	1.3 (-0.4 to 3.2)	3.4 (1.1 to 6.2)
Prothrombin time (seconds, range)	1,648 (29%)	14.1 (13 to 16.8)	12.0 (12.0 to 13.2)	13.2 (13.2 to 15.6)	14.4 (13.5 to 15.5)	14.1 (13.4 to 14.8)	15.8 (13.0 to 21.0)
Time to emergency department (minutes, range)	2,396 (42%)	56 (37-80)	62 (49 to 81)	47 (30 to 85)	27 (22 to 35)	*--*	63 (48 to 85)

The Amsterdam dataset did not record time from injury to emergency department arrival, and coagulation data in the German TR-DGU registry were recorded as Quick values for prothrombin time [39]. The Oslo dataset covers a 2-year period. TR-DGU, Trauma Registry of the Deutsche Gesellschaft für Unfallchirurgie.

**Figure 3:**
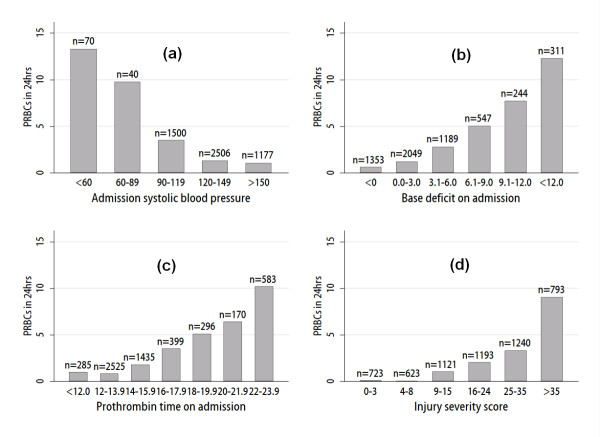
**Scatterplots showing admission parameters and injury severity associated with transfusion requirements**. Where covariates are missing for patient data, an average of imputed values has been substituted. **(a) **Packed red blood cells (PRBCs) transfusions by admission systolic blood pressure. **(b) **PRBC transfusions by admission base deficit. **(c) **PRBC transfusions by admission prothrombin time. **(d) **PRBC transfusions by injury-severity score.

Table 2 reports the regression coefficients from the logistic regression model. For the prediction of patients requiring massive transfusion, transformation toward a normal distribution for skewed continuous covariates was undertaken, as shown in column 1, Table 2. Log-odds and odds ratios for each variable are shown (log-odds can be more readily added together to calculate patient-specific probability of massive transfusion, and odds ratios are more meaningful for considering the impact of an individual predictor). The variables with the most weight in the model were systolic blood pressure (Figure 3a), base deficit (Figure 3b) and prothrombin time (Figure 3c). Age, penetrating injury, and time to emergency department were also identified as important dependent variables. Injury severity is known to be related to transfusion requirements (Figure 3d), but because accurate ISS scores are not directly available on admission, these measures were excluded from the final model, as shown. However, when a model including ISS was fitted, it was found that ISS was a significant predictor and gave more accurate predictions of massive transfusion (data not shown). For continuous variables, the odds ratios apply to a unit increase in the transformed variable (for example, √age). A patient's logit probability, A, of transfusion could be calculated by summing the intercept and appropriate log-odds ratios for their parameters by using Table 2. The probability of massive transfusion was then calculated from $\exp(\frac{A}{1+A})$.

**Table 2:** Regression coefficients from logistic regression model

	Log-odds ratio (SEM)	Odds ratio (95% CI)
√**age**	0.16 (0.05)	1.2 (1.1 to 1.3)
Ln (**time to emergency department**)	0.06 (0.17)	1.1 (0.8 to 1.5)
**Penetrating injury**	0.4 (0.24)	1.5 (0.9 to 2.4)
**Systolic blood pressure**	-0.02 (0.003)	0.98 (0.97 to 0.98)
ln(25^a ^+ **base deficit**)	5.48 (0.5)	240 (91 to 639)
1/(ln(**prothrombin time**^2^))	-26.7 (4.3)	2.5 × 10^-12 ^(5.3 × 10^-16 ^to 1.2 × 10^-8^)
Intercept	-16.7 (2.3)	-

^a^25 is an arbitrary constant added to base deficit to ensure positive values before logarithmic transformation.

**Figure 4:**
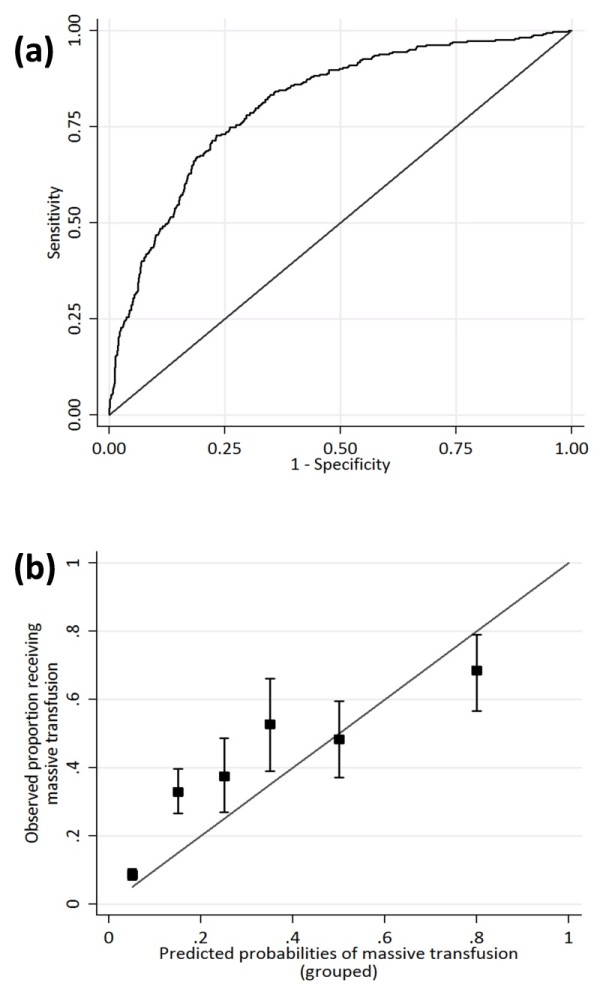
**Performance of the massive-transfusion prediction tool**. The performance of the model developed on non-German TR-DGU centers and validated on German TR-DGU registry data (see text). **(a) **Receiver operating characteristic plot. Area under the ROC curve, 0.81. **(b) **Calibration plot.

The receiver operating characteristic (ROC) curve is shown in Figure 4a and has an area under the curve (AUC) of 0.81, externally validated on the German TR-DGU data. This model performed less well at intermediate and higher probabilities of 10+ PRBC transfusions (Figure 4b). At a sensitivity of 90%, specificity for massive transfusion was only 50%, with 58% of patients correctly classified. For the internal validation (60 to 40 split), the identical set of variables was selected; in this case, the AUC was 0.89 (95% confidence interval, 0.87 to 0.92), with a specificity of 70% at 90% sensitivity. The model varied in performance when applied to specific trauma centers. At a sensitivity of 90%, the specificity varied from 48% (San Francisco) to 91% (Amsterdam). Complete data analysis was entirely consistent with the multiple imputation analysis in terms of parameter estimates and confidence intervals (CIs). The only difference was reflected in less-precise parameter estimates, as would be expected. Because validation was on the German TR-DGU centers, and these had no missing data, the inferences were very similar to those using multiple imputation.

## Discussion

This international multicenter study was conducted to evaluate the clinical applicability of massive transfusion as a concept in modern trauma care. The five trauma datasets represent a range of sizes and activities, which are likely to be generalizeable to many different trauma units worldwide. Any definition of massive transfusion should be useful in terms of its relevance to patient outcome. We have shown an association between transfusion and mortality, with a continuous increase in risk, and with a steeper increase in the lower ranges of the curve. We were not able to identify the traditional 10 units of PRBCs or any other specific threshold definition of massive transfusion, based on a mortality outcome. Patients receiving six to nine units of PRBCs had nearly 2.5 times the mortality of patients receiving none to five units. Management strategies targeted at patients receiving a threshold of 10 or more PRBC units will exclude a large proportion of patients receiving fewer transfusions but who still have a significant mortality. Research studies examining only massively transfused patients, according to this definition, will therefore exclude an important patient group. Moreover, therapeutic intervention studies will be confounded by any treatment effect that results in reduced PRBC requirements and therefore the inappropriate exclusion of patients from the study population. This may be one factor relevant to discussions about the internal validity of retrospective reports suggesting benefit with increased plasma and platelet transfusions in massively transfused patients [12-14,33-35].

The utility of the massive-transfusion concept may better apply for its therapeutic potential, and it may have a role in the activation of major hemorrhage protocols. Damage-control resuscitation strategies require early administration of blood-component therapy along with the first units of PRBCs [11], and attempts have been made to develop prediction algorithms for massive transfusion [15-20]. Our prediction model has been robustly validated across multiple centers, a larger sample size, and a wider geographic area, and uses variables that are potentially available soon after arrival in the emergency department. However, the performance of the model was only moderate, and the AUC of our tool of 0.81 is consistent with other prediction tools (0.68 to 0.85) [15-20]. Setting the sensitivity at a clinically useful threshold of 90% (at which 10% of actively bleeding patients will be missed initially), the tool has a specificity of only 50%. [15-20]. The consequences of lower specificity is the risk of inappropriate activations of transfusion protocols, wasting of blood products, and increased exposure of patients to adverse events related to transfusion. The potentially harmful effects of PRBCs in trauma patients, especially in relation to storage age, have been documented [36]. This will have increasing impact as protocols move toward much higher doses of plasma, platelets, fibrinogen, and cryoprecipitate.

One of the reasons for the difficulties in developing any models with high specificity and sensitivity is likely to be the heterogeneity in patient populations of trauma. Existing transfusion practices may also limit its utility in clinical practice. This study shows that the reliable prediction of massive transfusion from standard admission physiology alone is difficult. The components of the prediction model were heavily weighted toward systolic blood pressure, base deficit, and the prothrombin time, which are the main features driving the development of ATC [6].

The performance of the tool might be improved if a better near-patient measure of the severity of the coagulopathy were available (for example, functional tests of coagulation such as thromboelastometry or thromboelastography) [37,38]. Injury severity is also a strong dependent variable for the prediction of ATC and massive transfusion [6-9], but is not immediately available. Whether it is possible to develop an alternative but comparable measure for ISS that is available soon after admission remains unclear. Currently, no biomarkers of tissue injury are available, but such a rapidly available measure might also significantly improve prediction algorithms for ATC, massive hemorrhage, and patient care. Future work must look at these alternative approaches to developing a clinically useful prediction tool, because even across multiple datasets and with the application of several validation techniques, this study was not able to develop a reliable prediction tool.

Some limitations exist in this study. It is a retrospective review of registry data in which a variable proportion of records contained missing data, but this is inevitable to a degree in analyses of multiple registries. Multiple imputation assumes that missing data are random, having accounted for observed covariates, but this may not have been the case if variables that predict missing data were not recorded. However, the model performed well against the German TR-DGU data, which were more plentiful, indicating geographic transportability [30]. Entry criteria for the datasets were also recognized to be different. The San Francisco dataset included only patients with a higher-level trauma team activation, whereas the German TR-DGU included only patients with an ISS of 9 or higher. It was not possible to standardize the measurements of PT between the centers, as different thromboplastins were used, each with a different laboratory-specific Mean Normal Prothrombin Time (MNPT) and International Specificity Index (ISI), although in this study, the variations in reference ranges and results for PT were small, and the majority of results were normal or only marginally increased [39].

The mortality model may also be confounded because, as for patients dying within 24 hours, the rate of PRBC transfusion may have been higher than indicated in the data [40]. In addition, it is difficult to exclude an effect due to censoring for death, as some patients may die before sufficient time to receive blood. The rate of bleeding is not available from standard registry data but has been identified as an important confounder in the retrospective high-dose plasma studies [3]. Another limitation is the lack of information between centers on indications for transfusing PRBCs, the variation in transfusion practices, and the use of hemostatic drugs such as antifibrinolytics or even recombinant activated factor VIIa [41]. Massive transfusion not only is the result of a set of clinical parameters but it also is a function of the clinical response to them.

## Conclusions

In summary, current definitions of massive transfusion are not supported by clinical outcomes and are not useful for guiding management. Rather, mortality increases with each PRBC unit required, although not linearly. The robust prediction of massive transfusion from standard admission parameters remains difficult. The concept of massive hemorrhage may be more useful than is massive transfusion for modern trauma care. New approaches are required for the early diagnosis of patients with acute traumatic coagulopathy who are actively bleeding and will go on to require significant blood-component transfusions.

## Key messages

• Red cell requirements in trauma correlate with mortality.

• No clinically relevant threshold defines massive transfusion in terms of clinical outcomes.

• Red cell transfusion requirements cannot reliably be predicted on the basis of standard physiological variables available on admission.

• Attention should be focused on identifying patients with massive hemorrhage.

• New diagnostic modalities are needed for the early identification of acute traumatic coagulopathy.

## Abbreviations

ATC: acute traumatic coagulopathy; AUC: area under the curve; ISI: international specificity index; ISS: injury severity score; MNPT: mean normal prothrombin time; PRBC: packed red blood cell; PT: prothrombin time; ROC: receiver operating characteristic; TR-DGU: Trauma Registry of the Deutsche Gesellschaft für Unfallchirurgie.

## Competing interests

The authors declare that they have no competing interests.

## Authors' contributions

KB conceived the study, TM and TJ undertook the statistical analysis with SS and KB, and all other authors contributed to study design, data sharing, and writing of the manuscript.
